# Molecular measurement of *BCR-ABL *transcript variations in chronic myeloid leukemia patients in cytogenetic remission

**DOI:** 10.1186/1471-2326-10-7

**Published:** 2010-11-18

**Authors:** Mariana Serpa, Sabri S Sanabani, Pedro Enrique Dorliac-Llacer, Monika Conchon, Thales Dalessandro Meneguin Pereira, Luciana Nardinelli, Juliana Lima Costa, Mafalda Megumi Yoshinaga Novaes, Patricia de Barros Ferreira, Israel Bendit

**Affiliations:** 1 Department of Hematology, Faculty of Medicine, University of São Paulo, São Paulo, Brazil; 2 Cancer Institute of São Paulo, São Paulo, Brazil; 3 Clinical Immunology and Allergy Division, University of São Paulo, São Paulo, Brazil; 4 Department of translational medicine, Federal University of São Paulo, São Paulo, Brazil

## Abstract

**Background:**

The monitoring of *BCR-ABL *transcript levels by real-time quantitative polymerase chain reaction (RT-qPCR) has become important to assess minimal residual disease (MRD) and standard of care in the treatment of chronic myeloid leukemia (CML). In this study, we performed a prospective, sequential analysis using RT-qPCR monitoring of *BCR-ABL *gene rearrangements in blood samples from 91 CML patients in chronic phase (CP) who achieved complete cytogenetic remission (CCyR) and major molecular remission (MMR) throughout imatinib treatment.

**Methods:**

The absolute level of *BCR-ABL *transcript from peripheral blood was serially measured every 4 to 12 weeks by RT-qPCR. Only level variations > 0.5%, according to the international scale, was considered positive. Sequential cytogenetic analysis was also performed in bone marrow samples from all patients using standard protocols.

**Results:**

Based on sequential analysis of *BCR-ABL *transcripts, the 91 patients were divided into three categories: (A) 57 (62.6%) had no variation on sequential analysis; (B) 30 (32.9%) had a single positive variation result obtained in a single sample; and (C) 4 (4.39%) had variations of *BCR-ABL *transcripts in at least two consecutive samples. Of the 34 patients who had elevated levels of transcripts (group B and C), 19 (55.8%) had a < 1% of *BCR-ABL/BCR *ratio, 13 (38.2%) patients had a 1% to 10% increase and 2 patients had a >10% increase of RT-qPCR. The last two patients had lost a CCyR, and none of them showed mutations in the *ABL *gene. Transient cytogenetic alterations in Ph-negative cells were observed in five (5.5%) patients, and none of whom lost CCyR.

**Conclusions:**

Despite an increase levels of *BCR-ABL/BCR *ratio variations by RT-qPCR, the majority of CML patients with MMR remained in CCyR. Thus, such single variations should neither be considered predictive of subsequent failure and nor an indication for altering imatinib dose or switching to second generation therapy. Changing of imatinib on the basis of *BCR-ABL/BCR*% sustained increase and mutational studies is a prudent approach for preserving other therapeutic options in imatinib-resistant patients.

## Background

CML is a myeloproliferative disorder of blood stem cells [1]. The causative molecular defect is the BCR-ABL protein, which is encoded by the Philadelphia chromosome (Ph) [2]. This genetic anomaly arises from an exchange of genetic material between chromosomes 9 and 22, which results in the fusion of the breakpoint cluster region (*BCR*) and the Abelson leukemia virus (*ABL*) protooncogene [3,4]. The resulting gene encodes a constitutively active protein kinase that activates a number of proteins involved in cell-cycle regulation that hasten cell division and affect DNA repair [5-8]. The clinical course in CML is divided into a chronic phase (CP), accelerated phase (AP), and blast phase (BP) [2]. During CP, there is a gross expansion of the myeloid compartment, but the cells still retain the capacity to differentiate and function normally. During AP, the myeloid compartment undergoes transformation, with an increased number of immature myeloid cells within the bone marrow [2,9]. The terminal phase is transformation to acute leukemia [2,9]. The search for a specific inhibitor of the BCR-ABL tyrosine kinase have resulted in the identification of the specific inhibitor imatinib mesylate (STI571), which has now become the standard first-line therapy in patients with CP-CML [10,11]. Imatinib, also known as Gleevec^® ^(Novartis, Basel, Switzerland), is a selective inhibitor not only of ABL but also for Kit and PDGFR kinases and exerts significant antileukemic activity in the majority of CML patients. The efficacy of imatinib was defined by quantifying *BCR-ABL *transcripts to evaluate the residual disease burden in patients who attained a complete cytogenetic response (0% Ph positive chromosome in 20 metaphases) [12]. A > 3 log reduction in *BCR-ABL *fusion transcript levels (1000 fold reduction; 0.1% *BCR-ABL/BCR *ratio according to the international scale (IS)) from the baseline mean, defined as a MMR, is a favorable prognostic factor for the disease at any time during therapy. In term of treatment responses, it has been reported that this *BCR-ABL *kinase inhibitor produces a complete hematological response in 98% of patients, whereas CCyR occurs by 60-months follow-up in 87% of newly diagnosed patients with CML in CP, and between 40% and 60% in late CP [13-19]. According to the most recent results of the International Randomized Study of Interferon versus STI571 (IRIS) trial, the estimated overall survival for patients still on imatinib was 85% at 8 years, or 93% when only CML-related deaths or deaths prior to stem cell transplant were included. A total of 92% were free of disease progression. The risk of progressing to AP- or BP-CML was 0.9% in year 4 of treatment, 0.5% in year 5, 0% in years 6 and 7, and 0.4% in year 8. Among those who achieved CCyR, only 3% progressed during the 8-year follow-up [20]. Moreover, patients achieving MMR do even better, as no patient who achieved MMR at 18 months had progressed at 5 years [12,16]. Patients who did not obtain a complete hematologic response at 3 months or CCyR at 18 months were at increased risk of relapse [14,16]. Despite this breakthrough in the treatment of CML patients, up to 27% of the patients who achieved CCyR have been shown to subsequently lose their response and consequently fail to derive adequate or lasting clinical benefit because of intolerance and/or resistance [21-24]. The results published by Druker and co-workers demonstrated that patients who had CCyR or in whom leukemic burden of *BCR-ABL *had fallen by three orders of magnitude (3 logs) or more had a significantly lower risk of relapse than did patients without a CCyR [16]. A rise in *BCR-ABL *transcript levels detected during imatinib therapy for Ph positive CML patients who achieved CCyR is an alarming indicator of suboptimal response and should trigger a subsequent, more stringent, RT-qPCR assessment [25]. Together, these results have prompted researchers to investigate whether it is possible to distinguish CCyR patients at imminent risk of relapse from those likely to derive benefit from imatinib treatment. As a consequence, identification of those candidates with eventual molecular relapse early on would be useful to change the monitoring frequency and enable the use of alternative, more potent, second-generation TKI therapies that may be more effective. Molecular monitoring of the *BCR-ABL *transcript by RT-qPCR in patients with CCyR during treatment is arguably the single most important tool to evaluate the outcome and to assess the risk of impending relapse. Currently, a threshold of 5-fold to 10-fold (0.5 or 1 log) increase in *BCR-ABL *transcript has been proposed for molecular relapse. Against this background, we aimed to investigate whether serial monitoring of *BCR-ABL *by RT-qPCR, but not cytogenetic analysis, to measure minimal residual disease (MRD), performed throughout imatinib treatment in CP-CML patients having achieved CCyR and MMR, could be safely used to monitor patients and predict the probability of relapse within a clinically relevant time period and to guide therapy.

## Methods

### Patients

Between June 1998 and September 2008, 217 adult patients aged 16 to 75 with CML in CP undergoing treatment with imatinib 400 mg/day at our center were enrolled in this study at the time of diagnosis. Of these, 91 (41.9%) had reached both CCyR and MMR and were considered for inclusion in this study. Imatinib was introduced in our institution at the end of 2000; since then, patients with CML have been treated with this inhibitor every time the therapy-related toxicity allowed it. The CP was defined by the presence of < 15% blasts, < 20% basophils and < 30% blasts plus promyelocytes in both peripheral blood and bone marrow; a platelet count of at least 1 × 10^5 ^per cubic millimeter; and no evidence of extra medulary disease [15,26,27]. The median age of the patients at diagnosis was 45 years (range 16 to 75 years). Of the 91 subjects, 29 (31.9%) had been treated previously with interferon (IFN-α), and only 2 of them had achieved CCyR. In these two patients, the treatment was switched to imatinib due to IFN-α toxicity. The study cohort comprised 44 males and 47 females (Table 1). Tests of bone marrow morphology and cytogenetic studies were carried out at diagnosis, every 6 months until CCyR was achieved and then annually thereafter. Criteria for CCyR included morphologically normal bone marrow with complete disappearance of Ph-positive in at least 20 metaphases examined. Cytogenetic relapse was defined by the detection of one or more Ph-positive marrow metaphases and confirmed by a subsequent cytogenetic study. CCyR was considered durable if it lasted for at least 6 months. Patients were reported to have achieved MMR or CMR if their *BCR-ABL/BCR *ratios showed a reduction to 0.1% (≤ 3log) or 0.005% (≤ 4.5log), respectively, from a standardized baseline according to the IS [28] and confirmed in two subsequent samples. Numbers of *BCR-ABL *transcript were measured by RT-qPCR at 4 to 12 weeks beginning in February 2006. Variation levels of *BCR-ABL *were considered positive if samples showed an increase of more than 0.5% of transcripts, according to the IS, compared to that seen in early MMR. The cutoff of 0.5% was chosen with reference to the study by Mauro et al [29]. All patients initially received imatinib at an oral dose of 400 mg/day without concomitant chemotherapy.

**Table 1:** Patient's characteristics

Number of patients	91
Median age, years (range)	45 (16-75)
Male	44 (48.4)
Female	47 (51.6)
Prior therapy with interferon-α	29
Cytogenetic abnormalities in Ph-negative cells, no. (%)	5 (5.5%)
Time from initiation of imatinib to CCyR (months) Median (range)	7 (6-50)
Time from initiation of imatinib to MMR (months) Median (range)	18 (9-65)
Time from initiation of imatinib to CMR (months) Median (range)	33 (5-78)
Time from achievement of CCyR to last evaluation (months) Median (range)	57 (24-95)
Time from achievement of MMR to last evaluation (months) Median (range)	36 (19-46)
Time from achievement of CMR to last evaluation (months) Median (range)	22 (6-49)

### Cytogenetic investigation

Cytogenetic analysis of bone marrow was routinely performed using the Giemsa-Trypsin-Wright (GTW) stain banding technique [30]. At least 20 metaphases were analyzed for each sample.

### Samples and RNA isolation

A volume of 10 to 30 ml of fresh peripheral blood from each patient was collected in EDTA and treated with 0.144 M NH_4_Cl and, 0.01 M NH_4_HCO_3 _within 2 h of collection to lyse the red cells, as previously described [31]. Total leukocyte RNA was extracted by using RNAzol™(Invitrogen Life Technologies, San Diego, CA, USA) according to the manufacturer's instructions. cDNA was synthesized from 2 μg RNA using random hexamers as previously described [31].

### Measurement of *BCR-ABL *transcript numbers and mutational analysis

*BCR-ABL *and *BCR *transcripts in cDNA were measured by RT-qPCR using the procedure described by Branford *et al. *[32]. To minimize sampling error, the measurements of *BCR-ABL *and *BCR *transcripts were performed in duplicate. The copy numbers were calculated by comparison with the standard curve generated from serial dilutions of linearized plasmid containing the *BCR-ABL *and *BCR *inserts as described previously [32]. *BCR-ABL *mutation analysis was performed as described elsewhere [33].

### Statistical analysis

Comparisons of the *BCR-ABL/BCR *ratios at various time points during treatment and CCyR were performed by the nonparametric Kruskal-Wallis test. All analyses were performed using the SPSS software package (SPSS Inc., Chicago, IL, USA).

## Results

Of the 217 patients with CP-CML enrolled in this study, 91 (41.9%) had achieved CCyR and MMR after initiation of imatinib therapy and were included in the prospective monitoring study of MRD. Of these patients, CMR was achieved in 39 patients (42.8%). The median time from achievement of CCyR, MMR, and CMR to last follow-up evaluation (March 2010) was 57 months (range 24 to 95), 36 months (range 19 to 46), and 22 months (range 6 to 49), respectively (Table 1). The median time from starting treatment to achievement of CCyR, MMR, and CMR was 7 months (range 6 to 50), 18 months (range 9 to 65), and 33 months (range 5 to 78), respectively. Two patients already had CCyR with previous IFN-α treatment but were switched to imatinib because of intolerance of side-effects. Of the other 89 patients, 27 were initially assigned to IFN-α but had crossed over to imatinib due to an adverse event or because they did not achieve CCyR by the designated target dates. Overall, treatment with standard-dose imatinib produced an optimal response in 73 (80.2%) patients. All but one of these 73 patients never had a relapse, and their median follow-up period was 53 months (range 37 to 141). Of the other 18 (19.2%) patients, 10 (11%) had treatment failure and 8 (8.8%) had suboptimal response according to the definitions of failure of and suboptimal response to imatinib proposed by the European Leukemia Network [25]. None of these patients had genetic incidence of mutations conferring resistance to imatinib. Of the latter 18 patients, imatinib dose was escalated to 500 mg/day in 1 patient, to 600 mg/day in 14 patients and up to 800 mg/day in the other 3 patients. Following imatinib dose escalation, 12 patients did not achieve CCyR at 18 months of treatment and remained under the same treatment due to the unavailability of a second inhibitor at that time; however, they did respond at later times (up to 51 months). Five other patients had dose reduction to 300 mg/day because of intolerance or side-effects (four with neutropenia and one with congestive heart failure) and, as of this writing, are still in MMR (15 to 42 months). One patient who received further imatinib dose escalation to 800 mg/day required dose reduction to 400 mg/day because of higher hematological toxicity.

Based on sequential analysis of *BCR-ABL *transcripts, the 91 patients were divided into three subgroups. Subgroup A consisted of patients who had no variation on sequential analysis. Patients who had a single positive variation result obtained in a single sample were allocated to subgroup B. Subgroup C consisted of patients who had variations of *BCR-ABL *transcripts in at least two consecutive samples. As depicted in Table 2, 57 (62.6%) patients were assigned to group A, 30 (32.9%) to group B and 4 (4.39%) to group C. Of the 34 patients (group B and C) who had at least a single variation in *BCR-ABL *RT-qPCR, 19 (55.8%) had a < 1% of *BCR-ABL/BCR *ratio, 13 (38.2%) patients had a 1% to 10% increase and 2 patients had a >10% increase of RT-qPCR.

**Table 2:** Molecular, treatment with imatinib and cytogenetic assessment of clinical samples of CML patients studied

	No. of Patients	Group A (*n *57)	Group B (*n *30)	Group C (*n *4)
Variations in *BCR-ABL/BCR %*				
0.5-< 1	19	0	19	0
1-10	13	0	11	2
> 10	2	0	0	2
Imatinib dose during follw up until achievement of CCyR				
No change	68	45	21	2
Dose escalation	18	9	7	2
Dose reduction	5	3	2	0
Loss of CCyR	2	0	0	2
Alteration in Ph negative cells	5	4	1	0
Achievement of CMR	39	29	10	0

Only 4 patients (group C) had variations of BCR-ABL transcripts in consecutives samples. One of these four patients developed the M244V mutation. Despite having CCyR, the patient's imatinib was discontinued and dasatinib was started at a dose of 100 mg once daily and regained MMR in the last evaluation (March 2010). Another patient have to take more medications on a daily basis for sickle cell anemia and admitted to having missing some doses. During follow-up, the other two patients who had a >10% increase of RT-qPCR lost CCyR without evidence of mutation on the ABL kinase domain. Of these two cases, one had not achieved CCyR at 18 months while taking imatinib at 400 mg/day. The same patient achieved both CCyR and MMR after 26 months with an increase of imatinib to 800 mg/day; however, 4 months later the transcript levels had increased to 11.54% compared to baseline. As a result, treatment was switched to nilotinib 400 mg twice daily and further to bosutinib 500 mg/day, but without response. The possibility of non-compliance appeared inevitable and was discussed repeatedly, and finally admitted by the patient. The patient was on dasatinib 100 mg/day and had achieved CCyR after 4 months of therapy. The second patient achieved both CCyR and MMR after 6 and 18 months of therapy, respectively. The same patient maintained MMR for only 4 months, and molecular analysis of peripheral blood revealed a continuous increase of the *BCR-ABL *transcript from 0.08% to 1.8% and then to 3.6%. However, the latter patient had gastric intolerance of nausea and vomiting despite the use of antiemetic, which may have impaired drug absorption. As a consequence, imatinib was safely switched to nilotinib 400 mg twice daily, and the patient regained CCyR and MMR at 6 months.

As shown in table 2, CMR was achieved in 39 patients (29 in group A, 10 in group B, none in group C) and of them, only 9 patients (8 in group A and 1 in group B) have ongoing CMR. There was no significant difference between patients in group B in relation to CMR (P = 0.11). Of the other 30 patients who achieved CMR, 18 had *BCR-ABL/BCR *ratio increase from 0.005% to 0.01%, 7 had *BCR-ABL/BCR *ratio raised between 0.005% - 0.1%, and 5 had increased ratio greater than 0.5%. Of note, these variations were observed in some of the collected samples during follow-up period.

Previous exposure to IFN was documented in 29 patients, among whom 17 were in group A, 11 in group B (7 with *BCR-ABL *transcripts variation < 1%, and 4 with variations between 1-10%), and 1 in group C. Among the 39 patients with CMR, 12 were pretreated with IFN-α: eight in group A, and four in group B. There was no difference in response rate to imatinib between the group that were pre-treated with IFN-α, or those that did not have IFN-α before.

Our follow-up cytogenetic evaluations revealed five (5.5%) patients with chromosomal abnormalities in Ph-negative cells (Table 2). Of these, three patients (two in group A and one in group B) had developed trisomy 8, one patient had inv(9) (group A), and one had monosomy 7 (Group A). These cytogenetic alterations were transient in four patients (lasting 6 to 40 months), and none of the five patients lost CCyR.

## Discussion

Since the introduction of imatinib in 1998, there has been a significant decline of 30% in treatment-related mortality for patients exposed to therapy compared with a historical control group of CML patients who underwent stem cell transplantation prior to the availability of imatinib [34]. Following the introduction of imatinib as first-line therapy for patients with CML, CCyR has become a more frequent event in these patients (> 85%), and there has been a dramatic improvement of overall survival (98%) and progression-free survival (99%) with the achievement of CCyR after 18 months of treatment, compared with 76% overall and 87% progression-free survival, with partial cytogenetic response [16,35,36]. Several studies have reported that patients with both CCyR and MMR have lower rates of CCyR loss. Some reported higher event-free survival compared with those who achieved CCyR but not MMR [16,37-39]. Several researchers have found greater progression-free survival in patients with MMR [23,32,40], but others have not supported this observation [16,36,39]. The impact on overall survival has not been confirmed [36,38], but the change of treatment to a second-generation inhibitor soon after the loss of CCyR may explain this lack of relevance to overall survival.

In this report, we investigated the potential of RT-qPCR measurement of *BCR-ABL *transcript levels to predict cytogenetic relapse by monitoring the *BCR-ABL *transcript every 4 to 12 weeks throughout imatinib treatment in 91 CP-CML patients who had achieved CCyR and MMR. At least one variation in RT-qPCR and relapse developed at the molecular level were detected in 34 (37.3%) patients and only 4 (group C) had variations of *BCR-ABL *transcripts in consecutives samples. The raise in *BCR-ABL/BCR *ratio was common even in patients who had achieved CMR. The only two patients who had a > 10% increase, were also the only two who lost CCyR. Overall, these findings indicate that a rising trend of *BCR-ABL *transcripts is more meaningful than a single elevation. Of note, one of these patients appeared to have become refractory not only to imatinib but also to nilotinib and bosutinib. The patient was confronted with the results of the laboratory follow-up and then admitted therapy noncompliance. Thus, in addition to technical variation, drug resistance and treatment failure during imatinib therapy, a rise in *BCR-ABL *transcript may indicate inadequate imatinib dose, particularly a lack of patient compliance to the therapy [41-43]. In our follow-up study by RT-qPCR, none of the patients who achieved MMR for > 1 year had lost CCyR. Unlike previous studies, the molecular monitoring was performed in short time intervals between sample collections (every 4 to 12 weeks), so greater variations in the number of transcripts were observed.

Apart from our study, the use of *BCR-ABL *RT-qPCR to distinguish CCyR patients at imminent risk of relapse from those likely to derive benefit from imatinib treatment, has already been the subject of several studies [22,23,25,37,38,44-47]. For instance, Marin *et al. *[37] quantitated *BCR-ABL *expression by real-time PCR in peripheral blood in 161 patients who had initiated imatinib therapy early after diagnosis of CML in CP and who had achieved CCyR. The results of their study indicated that increases in the transcript levels that do not reach the MMR status have no prognostic value, while a two-fold increase in the transcript levels that do amount to MMR is a critical factor for predicting relapse. Palandri *et al. *[48] measured *BCR-ABL *levels by real-time PCR in peripheral blood in 130 patients with CML in CP who achieved CCyR with imatinib therapy after IFN-α failure, and they found that patients in unstable MMR, defined by transcript levels sometimes above the MMR (between 0.5 and 1log of the MMR), had a similar risk of losing CCyR as those who had never achieved MMR. The recent study by Kantarjian *et al. *[47] evaluated the clinical relevance of increased of *BCR-ABL *detected in at least two consecutive samples from 116 patients in stable CCyR on imatinib therapy for longer than 18 months. Contrary to the results reported by Palandri *et al*., Kantarjian *et al *found that most of the patients with increases in *BCR-ABL *remain in CCyR and that patients who had CML progression (9.5%) were those who either lost MMR or never had MMR and who had > 1 log increase of *BCR-ABL *transcript. Our results are thus consistent with those of Kantarjian *et al*.

The emergence of cytogenetic abnormalities in Ph-negative cells have been described to occur after the selective suppression of the predominant Ph-positive clones with imatinib in 10% of cases [49-53], but the cause and clinical significance of this phenomenon has not been fully clarified. The most frequent cytogenetic abnormalities are -Y, +8, -7 or 7q-, the first being reported as a common phenomenon in healthy men with advanced age [51,54]. Several studies have reported that most patients with these clonal chromosomal abnormalities do not evolve to myelodysplastic syndrome (MDS) or acute myeloid leukemia (AML) or progress to CML [38,49-51,55,56], and such cytogenetic alterations are often transient [50,51]. However, a few cases of evolution to MDS or AML have been reported and have been correlated with possible damage to the Ph-negative stem cell secondary to previous treatment as a result of a long disease process [55-57]. Generally, the presence of these cytogenetic abnormalities in Ph-negative cells provides a warning signal, as described by the European Leukemia Network [58]. In our study, only five patients (5.5%) developed cytogenetic abnormalities in Ph-negative cells, and none of them lost CCyR. Only one of them was in late CP (group A, trisomy 8) and had received IFN-α prior to initiation of imatinib, while the others were in early CP and started imatinib as a first-line therapy. One patient developed inv(9) which is considered a structural chromosomal variation [59]. It is possible that the emergence of clonal abnormalities could be a consequence of a long disease process or prior therapy negatively inhibiting normal hematopoiesis, thus providing the selective pressure for the outgrowth of a resistant Ph-negative clone. Alternatively, it could be a result of direct effect of imatinib.

Overall, our data are consistent with the findings of two recent studies [36,56] and lend further support to the new recommendations of the European Leukemia Network of performing cytogenetic evaluation either 1 year after obtaining CCyR or regularly if molecular monitoring is not available [58].

## Conclusions

In conclusion, fluctuation in *BCR-ABL/BCR *is a common event and most of them may be merely due to inadequate adherence, variability in the sampling or measurement procedure. Evidence from this work and other published studies demonstrate that most patients with CP-CML in MMR and CMR, even with variations in PCR, remain in CCyR. Thus, such single variations should neither be considered predictive of subsequent failure and nor an indication for altering imatinib dose or switching to second generation therapy. We would like to emphasize that changing of imatinib on the basis of sustained increase of *BCR-ABL/BCR *ratio and mutational studies is a prudent approach for preserving other TKIs therapeutic options in patients with imatinib resistance.

## Competing interests

The authors declare that they have no competing interests.

## Authors' contributions

MS was responsible of the clinical management of patients, acquisition of data, drafting the manuscript; SS was responsible of the scientific revision, discussion and editing of the manuscript; MC and TDMP were involved in clinical management of patients and interpretation of data; LN, JLC and MMYN carried out the molecular assays; PBF did the cytogenetic assay and analysis; PED was supervisor of clinical management of patients and interpretation of data. IB conceived of the design of the study and coordination. All authors read and approved the final manuscript.

## Pre-publication history

The pre-publication history for this paper can be accessed here:

http://www.biomedcentral.com/1471-2326/10/7/prepub
